# Implementation of two alcohol reduction interventions among persons with hazardous alcohol use who are living with HIV in Thai Nguyen, Vietnam: a micro-costing analysis

**DOI:** 10.1080/16549716.2020.1814035

**Published:** 2020-09-07

**Authors:** Natalie A. Blackburn, Vivian F. Go, Quynh Bui, Heidi Hutton, Radhika P. Tampi, Teerada Sripaipan, Tran V. Ha, Carl Latkin, Shelley Golden, Carol Golin, Geetanjali Chander, Constantine Frangakis, Nisha Gottfredson, David W. Dowdy

**Affiliations:** a Department of Health Behavior, University of North Carolina at Chapel Hill Gillings School of Global Public Health, Chapel Hill, NC, USA; b Department of Psychiatry and Behavioral Sciences, Johns Hopkins School of Medicine, Baltimore, MD, USA; c Department of Epidemiology, Johns Hopkins Bloomberg School of Public Health, Baltimore, MD, USA; d Department of Health, Behavior and Society, Johns Hopkins Bloomberg School of Public Health, Baltimore, MD, USA; e Department of Medicine, Johns Hopkins School of Medicine, Baltimore, MD, USA; f Department of Biostatistics, Johns Hopkins Bloomberg School of Public Health, Baltimore, MD, USA

**Keywords:** HIV, alcohol treatment, substance use, dissemination and implementation, low-middle-income countries

## Abstract

**Background:**

Hazardous alcohol use is detrimental to persons with HIV (PWH), impacting medication adherence and liver function, yet globally resources to target alcohol use behavior in this population are limited. Few studies have identified the costs of integrating alcohol reduction interventions into HIV care.

**Objective:**

To estimate the costs of implementing and delivering two evidence-based behavioral counseling interventions targeting hazardous alcohol use among persons with HIV and to estimate the costs of scale-up in ART clinics in Thai Nguyen, Vietnam.

**Methods:**

We undertook a micro-costing approach to determine the costs of delivering two adapted evidence-based interventions to reduce alcohol use: an intensive combined cognitive behavioral therapy and motivational enhancement therapy-informed intervention (CoI) and an abbreviated brief alcohol intervention (BI). A total of 294 participants with hazardous alcohol use were identified through a brief screening tool and received the CoI (n = 147) and the BI (n = 147) over 3 months. We estimated costs using time and motion studies, budget analysis, staff interviews, and participant questionnaires. Data were collected from 2016 to 2018 in VND and converted to USD.

**Results:**

The total cost of implementation and administration of the intervention to 147 participants receiving the CoI was $13,900 ($95 per participant) and to 147 participants receiving the BI was $5700 ($39 per participant). Implementation and startup costs including training accounted for 27% of costs for the CoI and 28% for the BI. Counselor costs accounted for a large proportion of both the CoI (41%) and the BI (30%).

**Conclusions:**

Implementing and delivering alcohol reduction interventions to people with HIV in Vietnam with appropriate fidelity is costly. These costs may be reduced, particularly counselor labor costs, by using an evidence-based brief intervention format. Future research should explore the budgetary impact of brief and combined interventions to reduce hazardous alcohol use, particularly among vulnerable populations.

## Background

Alcohol use is a major contributor to health problems globally. Excessive drinking, including hazardous drinking, has a substantial impact on morbidity and mortality in populations, resulting in downstream effects on work productivity and local economies [1]. Countries spend on average 1% of their gross national product addressing problems related to alcohol use [2,3]. Cognitive behavioral therapy (CBT) and motivational enhancement therapy (MET) are evidence-based interventions for reducing hazardous alcohol use and are demonstrated as cost-effective in high-resource countries [4]. Limited cost-effectiveness research exists in this area, in part, because the cost of these behavioral therapies in resource-constrained settings is not known [5–7].

In Vietnam alcohol use is normative, and hazardous alcohol use is pervasive [8,9]. Few alcohol reduction interventions have been delivered in Vietnam, and none have reported on the costs of implementation and delivery of services. Evidence-based behavioral interventions can be more resource intensive because one-on-one counseling is often required. Since counseling is a time-intensive activity and counselors must have specialized skills to provide behavioral interventions with high fidelity, this approach results in burdens for both patients and healthcare systems. For the patient, costs associated with travel and lost wages in order to attend appointments can have a negative economic impact. From the healthcare provider perspective, administration of CBT and MET requires additional resources to provide counselors with appropriate training. However, because hazardous drinking is widely accepted in Vietnam and, as a result, a lack of individual knowledge about the negative health consequences [8], an intensive intervention may be necessary to improve health outcomes.

Hazardous alcohol use, defined as more than 14 drinks per week for men and more than 7 drinks per week for women [10], impacts persons with HIV (PWH) disproportionately. Previously published research from northern Vietnam identified the need for alcohol treatment services and identified the barriers to medication adherence among PWH with hazardous alcohol use [8,11–13]. In 2015 only half of all PWH in Vietnam were receiving antiretroviral therapy (ART) [14]. Substance use (including alcohol use) may be one of the main drivers of this limited ART coverage [8]. Furthermore, once initiating ART, PWH must maintain high levels of adherence – a challenge that can exacerbate their health problems by excessive alcohol use. PWH who engage in hazardous alcohol have documented poor adherence to ART, often because of missed doses [15,16].

Competing health priorities limit the number of programs supported by government funds even when programs are shown to be effective. Without information about the cost of delivering alcohol interventions in Vietnam, policymakers are unlikely to scale up evidence-based alcohol reduction interventions, as such interventions may be perceived as diverting limited resources from existing health programs. Collecting data on the per-client costs associated with implementing and delivering an evidence-based alcohol reduction intervention among PWH, novel for this setting, would inform policy discussions of health programs and contribute to the information needed for disease control priority setting in this population [17].

From 2016 to 2018, the Reducing Hazardous Alcohol Use among ART Clients (REDART) was implemented in northern Vietnam with the objective to improve abstinence from alcohol among persons receiving antiretroviral therapy (ART) at seven ART clinics in Thai Nguyen, Vietnam. Participants were randomized to one of the three arms: combined intervention (CoI), brief intervention (BI), and standard of care (SOC). At 12-month follow-up, the authors identified a significant (p =.001) increase in percent days abstinent in both intervention arms as compared to the SOC with an increase of 15% (95% CI, 6%,24%). When comparing the two intervention arms at 12-month follow-up the authors found no difference in change in percent days abstinent from alcohol as well as no difference in their secondary outcome of a reduction in heavy drinking days (defined as four or more drinks per day for men and three or more drinks per day for women). Furthermore, they did not identify a difference in intervention effectiveness for those persons with a history of injection drug use. The percent of persons who were virally suppressed, defined as <20 copies/mL, at 12 months was also significantly (p = 0.014) higher for those who received the BI (89%) as compared to those who received the SOC (78%) [18].

Given this trial, our study objective was to estimate the costs associated with implementing two separate evidence-based alcohol reduction interventions among HIV-positive persons with hazardous alcohol use adapted for the Vietnamese setting and estimate the costs of possible scale-up throughout northern Vietnam. Because of both the lack of existing cost information in this setting and the need to collect cost data in as much detail as possible to inform future implementation, we undertook a micro-costing approach [19] in which we sought to determine as closely as possible the costs of individual goods and services in the administration of each of the intervention arms in their entirety. This is compared to the gross-costing (“top-down”) approach which can inform higher-level budgetary decisions but may be less useful in this setting because of the need to capture unit costs (for example, costs of implementation) that have not been previously described and may not be reflected in top-down budgets.

## Methods

### Study setting

We prospectively collected data on costs from 2015 to 2016 as part of a three-arm randomized control trial delivered from 2016 to 2018 in Thai Nguyen, Vietnam. Thai Nguyen, located 100 km north of the capital of Hanoi, is the ninth largest province in Vietnam. This semi-urban mountainous province has a population of 1.1 million and an economy based on the export of green tea and steel. Participants were residents of Thai Nguyen who were HIV-positive and attended one of the seven government-run antiretroviral treatment (ART) clinics in the province. Men screened eligible for the study by receiving a score of four or above and women screened eligible for the study by receiving a score of three or above on the brief Alcohol Use Disorders Inventory Test (AUDIT-C) (i.e. current hazardous alcohol use), a brief questionnaire that has previously been validated in Vietnam [20]. At baseline enrollment, participants in all three arms were comparable to demographic characteristics.

### Interventions costed

Individuals eligible for the trial were randomized to one of the three arms: combined intervention (CoI), brief intervention (BI), and standard of care (SOC). Each arm contained 147 participants. The SOC in Vietnam is a verbal referral to a substance use provider; given that this is the existing approach in the country it was not possible to identify the costs and thus is outside the scope of this analysis. The interventions are described in detail elsewhere [21]. Briefly, the CoI draws from motivational enhancement therapy (MET) and cognitive behavioral therapy (CBT) using a client-centered, motivational approach where clients focus on skills-building for alcohol use behavior change, including drinking refusal skills, skills to cope with and manage cravings and triggers, and developing positive thoughts and attitudes. The CoI included six individual face-to-face counseling sessions that were estimated to take 60–75 minutes each and occurring approximately 1 week apart. In addition, individuals had the option to attend three group sessions offered over the course of the six-week intervention; each of the group sessions emphasized role-playing using the MET/CBT informed-skills, consisted of four to six individuals, and lasted approximately 65 minutes each. The BI was adapted from an evidence-based US-based program known as Project TrEAT [22] and consisted of two individual face-to-face sessions that were estimated to take 30–45 minutes each plus two individual booster phone sessions lasting 5 to 15 minutes each. The face-to-face sessions for the BI occurred approximately 1 month apart, and phone sessions occurred two to 3 weeks after each face-to-face session resulting in a six-week intervention. The content of BI sessions includes a review of the client’s drinking patterns, feedback on known harmful effects of hazardous drinking, and alcohol use behavior change strategies informed by social cognitive theory [23].

We undertook a micro-costing approach that included the healthcare provider and participant perspectives. In order to identify costs associated with the delivery of these two interventions, we defined four categories for costs: implementation, counselor costs, participant costs, and overhead. Each of these cost categories is described in more detail below. Briefly, implementation costs are costs associated with the activities to put the intervention in place. Trainings and manuals were included in these costs. We also determined the supervisor to be part of implementation costs given her role in supporting the counselors to develop and maintain skills in intervention delivery. We defined counselor costs as those associated with the maintenance of the intervention including time waiting for participants and time in counseling sessions as well as administrative costs such as scheduling counseling sessions. Participant costs were defined as those costs borne by the participant including travel time to and from the clinic, time spent in sessions, and other costs to them as a result of attending sessions such as paying for childcare and meals while away from home. Lastly, we included overhead costs (utility bills, rental fees) as a separate category.

### Formative research for intervention approach

In order to deliver the intervention, several key steps were taken in the formative stage. In 2014, qualitative formative research was conducted to understand perceptions, local culture, and motivations for alcohol use among PWH receiving ART in Thai Nguyen [8]. Interviews were conducted with providers (n = 3) and persons receiving care in an ART clinic (n = 30). Community interviews were conducted with local restaurant and karaoke bar owners (n = 9) [8]. In order to determine the cost of implementing these two interventions in a real-world setting, we analyzed cost data for the main study and not for the formative work, as we did not feel this work would need to be repeated to implement the intervention.

The findings from the formative research informed the adaptation of manuals from a previously implemented evidence-based US-based study [22]. One intervention manual for the CoI and one intervention manual for the BI were developed by the US-based subject matter expert, incorporating comments from the US and Vietnam teams [21]. The development of the manuals and corresponding handbooks included design, logo creation, printing of notebooks for clients and counselors, and translation. The final CoI manual was about 100 pages, and the BI manual was about 35 pages. The corresponding handbooks for both interventions were 30 pages each. We did not include the costs of translation in our analysis as we determined that this was a one-time formative cost and thus would not be necessary for scale-up to other sites in Vietnam. Logo design, however, may differ depending on the in-country context and thus we chose to include it.

In conjunction with the development and completion of the manual, from 2015 to 2016, a series of training sessions were conducted to provide skills and knowledge to counselors in both the BI and CoI. The training of counselors administering the CoI required 13 days and for those counselors administering the BI required 6 days; preliminary trainings were delivered to both sets of counselors.

To implement the study, the team leased separate rooms that were adjacent to the regular clinic and furnished the room with equipment including a locked cabinet in which to keep the participant files.

One program coordinator coordinated both the CoI and the BI. The program coordinator’s three-month salary is included in the cost of implementation (3 months is the estimated time to train staff and implement each six-week intervention). Her duties, including facilitating training sessions and continuous administrative check-ins with staff on counseling sessions, were equally split between the CoI and the BI reflecting the division of cost between the two interventions.

### Operational costs

Operational costs included telecommunication, photocopying, paper, water for staff, and refreshments for participants during individual as well as group counseling sessions.

### Costing data collection

Time-and-motion data were collected from December 2016 to February 2017. Budgetary data were collected retrospectively. We also estimated costs of implementation, as described above. Finally, we evaluated costs from the participant perspective, including self-reported time traveling to and from the clinic separate from HIV appointments as well as lost wages due to attending clinic appointments.

Human resource costs required to deliver the intervention were estimated using time and motion studies [24] in which one counselor acted as an observer of another counselor for an 8-hour workday. Collected in 2016, observers recorded and coded counselor time spent on different activities, including administrative tasks such as scheduling sessions with participants, phone calls, waiting for participants, time in counseling sessions, and time spent preparing for counseling sessions. Each counselor was observed on three separate days to estimate day-to-day variability in time requirements. To estimate overhead costs, we worked with local clinic staff to complete a data collection instrument that included such items as electric bills, security costs, and rental of rooms in the clinic. Study staff then filled in these data collection instruments as well as individual staff salaries and details of the implementation process. Once we determined these costs, we divided overhead equally between the two intervention arms since the space, furniture, and operational costs were shared by all counselors and staff.

From March 2016 to March 2018 participant cost data were collected using an interviewer-administered questionnaire designed to estimate individual participant costs, based on the Stop TB Partnership’s ‘Tool to Estimate Patients’ Costs’ [25]. Questions administered to participants included details about transportation, food, accommodations, childcare, other out-of-pocket costs, and estimates of lost wages [see supplementary material]. Participants completed this questionnaire at baseline, 3 months, 6 months, and 12 months. Data from the questionnaire were then used to calculate total travel and incidental costs; participants in the CoI arm had six in-person individual sessions and three in-person group sessions to attend, resulting in nine trips while participants in the BI had two in-person individual sessions to attend and two sessions by phone, resulting in only two trips to the clinic.

### Scale-up analysis

In an *a priori* sub-analysis, we also considered the cost of implementing the CoI and BI beyond Thai Nguyen. The team held three separate Skype calls to discuss costs to consider for scale-up. Local staff identified four nearby provinces in northern Vietnam (Lạng Sơn, Bắc Cạn, Phú Thọ, and Bắc Giang) in which we had data on the number of ART clinics and the number of clients served within each clinic in those provinces. The team then consulted with Thai Nguyen study staff to estimate the personnel, administrative and other resources that would be required to scale the intervention to include these four provinces, in addition to Thai Nguyen, totaling five provinces. These costs included, for example, training of additional staff, obtaining administrative approvals, and delivery of the intervention. We assumed that each ART clinic in each province would require a dedicated part-time staff member (counselor or nurse), drawn from existing personnel, to administer either the CoI or the BI, and that 2 months would be required to find and train a new counselor (ongoing while working). We assumed that training a newly hired staff member would take 2 weeks (for the training alone) and would be directed by a full-time coordinator who would also manage logistics and perform site visits across the entire program. Additionally, we assumed that existing staff (counselors and supervisors) would need to spend 3 days per year on re-training activities to ensure high-quality performance and up-to-date skills. We then used those estimates (from the five provinces) to scale to 25 provinces reflecting approximately half the size of the northern region (58 provinces) of Vietnam.

All cost data were collected in the local currency, Vietnamese Dong (VND), and then converted to USA Dollar (USD) based on the 2015 currency conversion of 21,921 USD to VND; 2015 was the year in which training and manual development began.

## Results

Details of the full trial sample have been published elsewhere [18]. A brief list of demographic information about the enrolled sample is presented in Table 1. The median age of those enrolled was 40 years (IQR 36–44), and nearly all were men (97%). The majority were married (69%) and held less than a high school diploma (79%). A little over half reported working full time (54%), and almost one-third of those enrolled reported a history of drug treatment (30%). A large portion of those enrolled also reported a history of injection drug use (81%) indicating a unique population with multiple types of substance use.

**Table 1.** Characteristics of participant population at baseline.

		Enrolled in Study (n = 440)
Characteristics		Median (IQR^a^) or n (%)
Age (years)		40 (36–44)
Males		426 (97%)
Education		
	Less than high school diploma	348 (79%)
	High school complete	51 (12%)
	Technical school (incomplete)	2 (1%)
	Technical school (complete)	25 (6%)
	College (incomplete)	2 (1%)
	College (complete)	13 (3%)
Marital Status		
	Single	66 (15%)
	Married	305 (69%)
	Widowed	12 (3%)
	Divorced	23 (5%)
	Separated	21 (5%)
	Living with partner but not married	13 (3%)
Employment Status		
	Working full time	238 (54%)
	Working less than full time	119 (27%)
	Unemployed but seeking work	41 (9%)
	Unemployed not seeking work	41 (9%)
	Retired	1 (0.2%)
Self-Reported General Health (0–100)		80 (70–90)
Recent (last 3 months) homelessness		6 (1%)
History of Drug Treatment^b^		130 (30%)
History of Injection Drug Use		356 (81%)

^a^Inter-quartile range.

^b^1 refusal.

Table 2 presents the economic costs of each intervention arm. The total economic cost of the CoI was 13,905 USD for 147 participants ($95 per participant). The total economic cost of the BI was 5716 USD for 147 participants ($39 per participant). Two full-time counselors were required for each intervention.

**Table 2.** Economic costs of each intervention arm by category.

		Intervention				
		*Combined Intervention*			*Brief Intervention*	
Category		$	%		$	%
Implementation						
	Manuals	616 USD	4%		511 USD	9%
	Training sessions	2,548 USD	18%		502 USD	9%
	Coordinator (3 months)^a^	616 USD	4%		616 USD	11%
	**Sub-total**	**3,780 USD**	**27%**		**1,629 USD**	**28%**
Counselor Costs (two counselors per arm)						
	Wait	230 USD	2%		262 USD	5%
	In-Person Individual Sessions	3,784 USD	27%		903 USD	16%
	Group Counseling	1,182 USD	9%		N/A	N/A
	Phone Counseling	N/A	N/A		144 USD	3%
	Administrative Tasks	158 USD	1%		135 USD	2%
	Break	267 USD	2%		244 USD	4%
	**Sub-total**	**5,621 USD**	**40%**		**1,688 USD**	**30%**
Participant Costs (total)						
	In-Person Individual Sessions	728 USD	5%		187 USD	3%
	Group Counseling	455 USD	3%		N/A	N/A
	Phone Counseling	N/A	N/A		37 USD	1%
	Travel (round trip)	1,172 USD	8%		261 USD	5%
	Incidentals	302 USD	2%		67 USD	1%
	**Sub-total**	**2,657 USD**	**19%**		**552 USD**	**9%**
Overhead (3 months)^a^						
	Room Rental	109 USD	1%		109 USD	2%
	Operations	233 USD	2%		233 USD	4%
	Transportation	821 USD	6%		821 USD	14%
	Furniture^b^	684 USD	5%		684 USD	12%
	**Sub-total**	**1,848 USD**	**13%**		**1,848 USD**	**32%**
**Grand Total**		**13,905 USD**	**100%**		**5,716 USD**	**100%**

^a^3 months is the length of the intervention for a participant.

^b^Furniture is a one-time fee.

### Implementation activities

The implementation of each intervention arm constituted a large proportion of the overall costs with implementation costs accounting for 27% ($3784) of the costs of the CoI and 28% ($1629) of the costs for the BI. Additionally, the CoI had greater training costs ($2548) than the BI ($502); this is in part due to the size of the manual required for counselors and the added hours of skill-building in counselors required to deliver the six-session individualized counseling sessions.

### Overhead costs

Overhead costs accounted for 13% of costs associated with the CoI ($1848) and 32% of costs associated with the BI ($1848).

### Counselor activities

The counselor labor costs for the CoI accounted for more than one-third of the overall cost (40%) while the counselor labor costs for the BI accounted for more than one-fourth (30%). Counselors for the CoI spent a median time of 52 minutes (48–60) per session in individual counseling sessions and a median time of 65 minutes per session in group sessions, whereas counselors for the BI spent a median time of 50 minutes (44–73) per individual counseling session and a median time of 8 minutes (8–12) on phone counseling sessions. Furthermore, counselors in the CoI received a monthly salary of 8,500,000 VND ($390) as compared to counselors in the BI who received a monthly salary of 7,000,000 VND ($320) reflecting the additional training they received for a more complex intervention. Counselors for the CoI spent a median 19 minutes (10–35) waiting for participants whereas counselors for the BI spent a median 29 minutes (13–48) waiting for participants.

### Participant activities

Participants self-reported a mean weekly salary of 830,000 VND or 38 USD; this figure included 19% of participants who reported being unemployed or retired. The greatest cost to participants was travel; a round trip to the clinic was estimated to take an average of 1 hour and result in lost wages of 16,400 VND ($0.75). The cost to the participant of the in-person individual sessions accounted for 5% of the total cost of the CoI and 3% of the BI. Incidentals (e.g. food, lodging, childcare to attend clinic visits) accounted for 2% of costs in the CoI and 1% of costs in the BI.

### Sub-analysis – proposed scale-up

We completed a sub-analysis from the healthcare provider perspective in order to determine how the per-person cost to deliver each intervention might be impacted by scaling up to more provinces. To scale up the CoI to five provinces in northern Vietnam we estimated a total cost of 139,198 USD (Table 3). This would result in 2,320 persons completing the CoI or a cost of 60 USD per participant. To scale up the BI to five provinces in northern Vietnam, we estimated a provider cost of 93,023 USD (Table 3). This would result in 6,960 persons completing the BI or a cost of 13 USD per participant. The cost to scale the CoI to 25 provinces would be 830,732, USD resulting in 14,720 persons completing the CoI at a cost of 56 USD per participant. The cost to scale the BI to 25 provinces would be 537,763, USD resulting in 44,160 persons completing the BI at a cost of 12 USD per participant.

**Table 3.** Scale-up of each intervention arm by category.

		Combined Intervention			Brief Intervention		
Number of Provinces^a^		1	5	25	1	5	25
Implementation	Manuals^b^	$308	$8,930	$56,659	$255	$7,409	$47,006
	Training sessions^c^	$1,459	$42,322	$268,528	$311	$9,029	$57,288
	Coordinator	$2,463	$12,317	$61,586	$2,463	$12,317	$61,586
	**Sub-total**	**$4,231**	**$63,569**	**$386,772**	**$3,030**	**$28,755**	**$165,880**
Counselor Costs (annual)							
	Number of Counselors	1	29	184	1	29	184
	Sessions^d^	$1,544	$46,655	$296,019	$1,228	$35,618	$225,990
	Administrative Tasks	$79	$2,286	$14,505	$68	$1,963	$12,458
	**Sub-total**	**$1,623**	**$48,941**	**$310,524**	**$1,296**	**$37,581**	**$238,447**
Overhead (annual)							
	Room Rental	$438	$2,190	$10,949	$438	$2,190	$10,949
	Operations	$931	$4,653	$23,266	$931	$4,653	$23,266
	Transportation	$3,285	$16,423	$82,114	$3,285	$16,423	$82,114
	Furniture^e^	$684	$3,421	$17,107	$684	$3,421	$17,107
	**Sub-total**	**$5,337**	**$26,687**	**$133,436**	**$5,337**	**$26,687**	**$133,436**
**Grand Total**		**$11,191**	**$139,198**	**$830,732**	**$9,664**	**$93,023**	**$537,763**

^a^Assumes one clinic in one province (Thai Nguyen); in the 5 provinces we selected there are 29 clinics; in the 25 provinces in the northern province, there are 184 clinics (these are not evenly distributed across each province).

^b^Manual cost includes logo design, printing but does not include translation costs.

^c^Assumes two weeks (10 working days) of initial training plus 3 days of ‘booster’ training per year per counselor.

^d^Assumes 15 sessions per week for the combined intervention and 20 sessions per week for the brief intervention.

^e^Furniture is a one-time fee.

## Discussion

In this comprehensive, ingredients-based costing of alcohol reduction interventions nested within a randomized trial in northern Vietnam, we found that the overall cost to deliver the CoI to 147 participants over a three-month period was 13,900 USD ($95 per participant), and the overall cost to deliver the BI to 147 participants over the same time period was less than half or 5,700 USD ($39 per participant) (Table 2). Counselor costs for the CoI were close to four times that of the BI ($5600 vs. 1700 USD); the proportional difference in participant costs was even greater with those receiving the CoI reporting costs nearly five times higher than those receiving the BI ($2700 versus 550 USD). These findings may help ART and other health programs understand the likely costs of alcohol reduction interventions among PWH in settings similar to semi-urban Vietnam.

Participants who received the CoI spent more on travel to access the intervention ($1200) compared to those receiving the BI ($260). Similarly, implementation costs and counselor costs were much higher for the CoI because the intervention is more intensive (six in-person individual sessions plus three in-person group sessions, versus two in-person individual sessions and two phone sessions for the BI) and thus requires more time and effort in both implementation and delivery. A high proportion of the costs for both the CoI and the BI was related to implementation, which reflects the amount of time required for training and preparation of the intervention and the role of the supervisor in facilitating the intervention introduction. As a result, implementation costs accounted for more than a quarter of the costs of the CoI (27%) and more than a quarter of the costs of the BI (28%).

The proportion of costs dedicated to counselors reflects the length of the intervention being delivered. The costs of brief interventions (defined as fewer than four contacts with a patient) in high-resource settings to reduce alcohol use have been frequently evaluated in the literature [26]. Brief interventions are ideal for primary care and emergency care settings because of the ability to deliver them in shorter time frames, thus limiting the burden on busy providers and reducing the likelihood that patients will be lost to follow-up between sessions [27]. By contrast, the investment in staff training and infrastructure for the CoI, including a dedicated person responsible for administering the counseling sessions, may make it more sustainable than the brief intervention in the long term, despite its substantially greater cost.

Future research should seek to understand the barriers to implementation of behavioral health interventions in HIV clinic settings. Despite a wealth of evidence from randomized control trials on the effectiveness of alcohol interventions [28,29], limited research has been completed in real-world settings with competing health priorities. Costs may play a role in implementation decisions, but other factors (e.g. logistical feasibility, political will) play an important role as well. Future studies could also collect data on the community-level impact of hazardous alcohol use (and its mitigation) among persons with HIV in Vietnam. PWH with hazardous alcohol use are more likely to have poor medication adherence and difficulties with maintenance in care – such health issues are likely to impact not only the healthcare system but an individual’s surrounding community. Additionally, given the changing landscape of both need and delivery related to HIV services (e.g. expanded substance treatment) in Vietnam, it is important to get a full picture of the cost burden of new programs (such as those for hazardous alcohol use reduction) as funding infrastructure changes. For example, one concern for the future of substance use treatment in ART clinic settings is the transition of many patients from receiving treatment at peripherally placed ART clinics to the hospital setting as a result of the reduction in international donor support [30]. The hospitals are busier than the stand-alone ART clinics, and it may be difficult for interventionists to find a space in hospitals where a counselor could conduct individual one-on-one counseling sessions. One benefit, however, may be that co-located services may allow for participants to receive care for multiple health issues at the appointment, and thus reducing trips to and from the clinic. Overall, this transition may have important cost implications for patients, counselors, and overseers of such programs.

There are limitations to our analysis. First, a true societal perspective would capture additional costs, such as the cost impact on family and community members if an individual commits a crime or cannot work because of alcohol use [31]. Such data could provide greater detail to decision-makers about the impacts of an alcohol reduction program more broadly in the community; by not including these potential economic savings, we may overestimate the societal costs of alcohol reduction. Additionally, our data on participant-level costs were collected via self-report rather than direct observation, thereby introducing inherent biases that can occur when individuals are reporting lost wages and other burdens they face when accessing care. Individuals may have been less inclined to report burdens they experienced when accessing care out of fear of seeming ungrateful, as any expression of disappointment or discomfort may be viewed as disrespectful to the physician or healthcare provider. Nevertheless, while potentially biased, we believe that the incorporation of patient costs is essential to provide a complete picture of the relative costs of these two interventions.

Overall the cost of the CoI was more than twice the cost of the BI, primarily reflecting additional costs for implementation and counselor and participant time. The cost to participants was also noteworthy given the expectations to attend appointments and arrange transportation to access their care. A scale-up of these alcohol reduction interventions beyond one clinic in Thai Nguyen may be feasible and could distribute the costs of implementation more broadly, thereby modestly reducing the per-person cost of the intervention. Behavioral interventions to reduce alcohol use that are delivered in settings where participants are already receiving care for HIV are ideal for convenience but do require considerations of space, staff sharing, and other unexplored barriers.

## Conclusion

To facilitate the delivery of an alcohol reduction behavioral program in an HIV clinic setting, an understanding of implementation and maintenance costs is required. This research can help decision-makers better understand the per-patient and component costs of implementing and delivering structured alcohol reduction interventions among people living with HIV in Vietnam and other similar settings. The global impact of alcohol use will strain economies and burden resource-constrained settings with competing health priorities. How we address this burden will partly depend on how well we understand the costs associated with doing so.

## Supplementary Material

Supplemental Material

Supplemental Material

## Paper context

Hazardous alcohol use has detrimental effects on persons with HIV. Behavioral interventions targeting alcohol use are effective in reducing hazardous drinking. However, the resources needed for these interventions are not typically available in HIV care settings. This study assesses the costs of delivering two behavioral interventions in the ART clinic setting in Vietnam by delineating the activities impacting implementation and delivery budgets. These data can inform the scale-up of alcohol reduction interventions within resource-constrained health systems.
