# Supplementary material for: Implementation of two alcohol reduction interventions among persons with hazardous alcohol use who are living with HIV in Thai Nguyen, Vietnam: a micro-costing analysis
Source: Glob Health Action. 2020 Sep 7;13(1):1814035. doi: 10.1080/16549716.2020.1814035 (PMC7781886; doi:10.1080/16549716.2020.1814035)
Supplement: Supplemental Material [file ZGHA_A_1814035_SM3745.docx]

Additional File 1. Modified Tool to Estimate Patient Costs

C1. Currently, how much money on average are you making weekly? (This can include salary, traded goods, etc.) ___ ___ ___ ___ ___ ___ Amount in Dong

C2. Over the past 6 months, how many times have you had to come into a clinic for HIV care? ___ ___ Number of visits

C3. Think back to a regular visit where you received HIV care, how long did it take you for the one way trip to the clinic? (Starting from where a participant usually comes from for a visit) ___ ___ Minutes ___ ___ Hours

C4. How did you get to the clinic? [INTERVIEWER: MARK ALL THAT APPLY] (0) Walk (1) Bicycle (2) Personal vehicle/car (3) Motorbike (4) Bus (5) Taxi (6) Xeom (7) Other, Specify: __________________________

C5. How far did you have to travel to get to the clinic? ___ ___ ___ ___ ___ Distance in kilometers [if less than 1 km, then format as “00.XX]

C6. How much in transport costs did you spend to get to the clinic? ___ ___ ___ ___ ___ ___ Amount in Dong

C7. How much in other costs, such as food, lodging, or child care did you spend to get to the clinic? Think of everything that you spent the day that you came to clinic, and add up all money, traded goods, etc., that you ended up spending that you would not have spent if you did not have to come to clinic. ___ ___ ___ ___ ___ ___ Amount in Dong

C8. From the time you left home to the time you returned home, how much time total did you spend on the clinic visit? ___ ___ Minutes ___ ___ Hours

C9. If you had been working during that time, how much money do you think you would have made? ___ ___ ___ ___ ___ ___ Amount in Dong

C10. Over the past 6 months, how many times have you had to be hospitalized due to your HIV infection? ___ ___ Number of visits. If 0, skip to D1

C11. On average, how much time did you spend in the hospital? ___ ___ Minutes ___ ___ Hours ___ ___ Days
